# Racial and ethnic disparities in a state‐wide registry of patients with pancreatic cancer and an exploratory investigation of cancer cachexia as a contributor to observed inequities

**DOI:** 10.1002/cam4.2180

**Published:** 2019-05-09

**Authors:** Jennifer B. Permuth, Ashley Clark Daly, Daniel Jeong, Jung W. Choi, Miles E. Cameron, Dung‐Tsa Chen, Jamie K. Teer, Tracey E. Barnett, Jiannong Li, Benjamin D. Powers, Nagalakshmi B. Kumar, Thomas J. George, Karla N. Ali, Tri Huynh, Shraddha Vyas, Clement K. Gwede, Vani N. Simmons, Pamela J. Hodul, Estrella M. Carballido, Andrew R. Judge, Jason B. Fleming, Nipun Merchant, Jose G. Trevino

**Affiliations:** ^1^ Department of Cancer Epidemiology Moffitt Cancer Center Tampa Florida; ^2^ Department of Gastrointestinal Oncology Moffitt Cancer Center Tampa Florida; ^3^ Division of Behavioral Health Idaho Department of Health and Welfare Boise Idaho; ^4^ Department of Diagnostic Radiology Moffitt Cancer Center Tampa Florida; ^5^ Department of Cancer Imaging & Metabolism Moffitt Cancer Center Tampa Florida; ^6^ Department of Surgery, Division of General Surgery University of Florida Health Sciences Center Gainesville Florida; ^7^ Department of Biostatistics and Bioinformatics Moffitt Cancer Center Tampa Florida; ^8^ School of Public Health University of North Texas Health Science Center Fort Worth Texas; ^9^ Department of Medicine University of Florida Health Sciences Center Gainesville Florida; ^10^ Department of Health Outcomes and Behavior Moffitt Cancer Center Tampa Florida; ^11^ Department of Physical Therapy University of Florida Gainesville Florida; ^12^ Department of Surgical Oncology, Sylvester Comprehensive Cancer Center University of Miami Miller School of Medicine Miami Florida

**Keywords:** cachexia, biomarkers, incidence, mortality, pancreatic cancer, racial disparities

## Abstract

Pancreatic cancer (PC) is characterized by racial/ethnic disparities and the debilitating muscle‐wasting condition, cancer cachexia. Florida ranks second in the number of PC deaths and has a large and understudied minority population. We examined the primary hypothesis that PC incidence and mortality rates may be highest among Black Floridians and the secondary hypothesis that biological correlates of cancer cachexia may underlie disparities. PC incidence and mortality rates were estimated by race/ethnicity, gender, and county using publicly available state‐wide cancer registry data that included approximately 2700 Black, 25 200 Non‐Hispanic White (NHW), and 3300 Hispanic/Latino (H/L) Floridians diagnosed between 2004 and 2014. Blacks within Florida experienced a significantly (*P* < 0.05) higher incidence (12.5/100 000) and mortality (10.97/100 000) compared to NHW (incidence = 11.2/100 000; mortality = 10.3/100 000) and H/L (incidence = 9.6/100 000; mortality = 8.7/100 000), especially in rural counties. To investigate radiologic and blood‐based correlates of cachexia, we leveraged data from a subset of patients evaluated at two geographically distinct Florida Cancer Centers. In Blacks compared to NHW matched on stage, markers of PC‐induced cachexia were more frequent and included greater decreases in core musculature compared to corresponding healthy control patients (25.0% vs 10.1% lower), greater decreases in psoas musculature over time (10.5% vs 4.8% loss), lower baseline serum albumin levels (3.8 vs 4.0 gm/dL), and higher platelet counts (332.8 vs 268.7 k/UL). Together, these findings suggest for the first time that PC and cachexia may affect Blacks disproportionately. Given its nearly universal contribution to illness and PC‐related deaths, the early diagnosis and treatment of cachexia may represent an avenue to improve health equity, quality of life, and survival.

## 1 INTRODUCTION

Pancreatic ductal adenocarcinoma, commonly known as pancreatic cancer (PC), is currently the third (soon to be second) leading cause of cancer‐related deaths in the United States (US) and has a five‐year relative survival rate of 9%.^1^, ^2^ Startling racial/ethnic disparities exist for PC. Blacks have 50%‐90% higher incidence and mortality rates than Non‐Hispanic Whites (NHW) and Hispanic/Latinos (H/L).^3^, ^4^, ^5^, ^6^, ^7^, ^8^, ^9^ National and regional databases including the Surveillance, Epidemiology, and End Results Registry (SEER) show that compared to NHW, Blacks with PC generally are (a) diagnosed at a younger age; (b) diagnosed at a more advanced stage; (c) more likely to be socioeconomically disadvantaged; (d) less likely to undergo surgery or chemotherapy; and (e) more likely to have worse overall survival.^3^, ^4^, ^5^, ^6^, ^7^, ^8^, ^9^, ^10^ Higher PC burden among Blacks has been attributed to risk factors such as tobacco exposure, obesity, and diabetes in some studies^11^ but not others.^4^ Furthermore, nonmodifiable factors such as genomics, epigenetics, and the microbiome have also been identified as potential contributors to PC disparities in Blacks.^12^, ^13^ From a socioeconomic standpoint, Blacks are less likely than other racial/ethnic groups to be insured, married, referred to PC specialists, or diagnosed/treated at high‐volume hospitals.^7^, ^14^, ^15^, ^16^ To date, most PC disparities research^3^, ^4^, ^5^, ^6^ has not included data from Florida, which is the third most populous state, has the highest percentage of people over 65, and is home to 8% of all Blacks and 9% of all H/L in the US.^17^ This is significant because Florida ranks second in lives lost to PC annually; in 2019, 3490 (7.8%) of 45 000 cancer‐related deaths expected in Florida will be due to PC.^1^ Due to these statistics and the fact that under‐treatment of and higher mortality from PC has been reported in rural geographic regions of Florida,^18^ Florida is a prime location for studying PC health disparities.

In addition to epidemiologic, socioeconomic, and geographical variables contributing to disparities, it is also critical to consider and evaluate modifiable biological factors that may underlie disparities and serve as targets for intervention. An understudied biological attribute of PC is cancer cachexia, an often progressive condition characterized by pathological loss of striated muscle (skeletal and cardiac) and fat stores, manifesting in the cardinal features of emaciation, weakness affecting functional status, impaired immune function, metabolic dysfunction, and poor quality of life (QoL).^19^, ^20^ The complex syndrome of cancer cachexia substantially contributes to cancer‐related morbidity and mortality and is an independent predictor of shorter survival, increased risk of treatment failure, and toxicity in the PC patient population.^19^, ^20^ In fact, in a large cohort of PC patients evaluated using the prospectively collected cancer registry at the Kaiser Permanente Medical Center, cachexia and not obesity was independently associated with worse survival.^25^ Cachexia, which affects 80% of patients with PC, is a continuum with three stages, all associated with varying degrees of inflammatory, immunologic, and metabolic changes: pre‐cachexia which involves anorexia with <5% weight loss; cachexia, when >5% weight loss has occurred; and refractory cachexia, the most advanced and irreversible stage that culminates with loss of physical function and death.^20^ Given that a high proportion of PC cases have at least pre‐cachexia at diagnosis, opportunities exist to minimize or delay cachexia progression prior to clinical debilitation, improve QoL, and enhance survival using approaches that incorporate nutritional support and novel targeted anti‐cachexia therapies.^20^, ^26^

Several blood‐based biomarkers of cancer cachexia exist, including serum albumin (a surrogate marker of malnutrition) and platelets (a surrogate marker of inflammatory and immunologic responses to tumors); decreased serum albumin levels and high platelet counts have been shown to help identify cachexia and predict poor outcomes.^27^, ^28^ Myopenia, or clinically relevant muscle wasting,^37^ is a radiologic marker of cachexia measured from routine computed tomography (CT) image analysis at the third lumbar vertebra (L3), a validated region for body composition analysis which is the gold standard for body composition analysis.^27^, ^38^, ^39^ We previously demonstrated that a measure of myopenia, the psoas muscle index (PMI), is a powerful predictor of survival in PC patients undergoing surgery^40^; low PMI correlated strongly with low preoperative serum albumin levels and poor survival. Given that the prevalence and consequences of muscle wasting are higher in Blacks than NHW^41^, ^42^ and low serum albumin levels and high platelet counts are surrogate markers of cachexia,^36^, ^40^ we have suggested that biologic correlates of PC‐induced cachexia may be more common among Black PC patients and possibly account for some degree of disparities in outcomes.

In this study, we used publicly available data to examine PC incidence, mortality rates, and characteristics of patients diagnosed and treated for PC. As medical images and laboratory values for a diverse cohort of PC cases are not available in publicly available datasets, we leveraged data from two large and geographically separate Florida Cancer Centers to evaluate correlates of cachexia and focused on Blacks and NHW, the two racial groups with the highest PC burden.

## 2 MATERIALS AND METHODS

### 2.1 Analyses using publicly available statewide data

We conducted a retrospective, case‐only descriptive analysis of data from cancer registries and hospitals in Florida. Florida's Statewide Cancer Registry, the Florida Cancer Data System (FCDS), has received the NAACCR Gold Standard Award, highlighting the quality, completeness (95% or higher in terms of case ascertainment), and timeliness of data submissions.^43^ Using the publicly available FCDS Incidence/Mortality Rates Inquiry Tool,^44^ mean age‐adjusted PC incidence and mortality rates for 2004‐2014 were estimated for the 31 200 Floridians diagnosed with PC during this timeframe (9% Black, 81% NHW, and 10% H/L). We estimated overall rates and stratified by gender and county. Chi‐squared tests were used to estimate differences between racial/ethnic groups at a level of statistical significance of *P* < 0.05.

Since the on‐line incidence/mortality tool available through FCDS does not provide data on the volume and racial/ethnic distribution of PC cases diagnosed and treated at individual Florida hospitals, we complemented the publically available aggregate FCDS data with 2012‐2014 inpatient hospital discharge data from the Florida Agency for Health Care Administration (AHCA).^45^ Individuals age 18 and above with a primary discharge diagnosis of PC and all stages of disesase (ICD‐CM9 codes 140.0‐239.9 plus V codes for chemotherapy and radiation) were included. Inpatient data from all quarters of 2015 were not available at the time of analysis and therefore are not presented.

### 2.2 Cachexia‐related analyses for PC cases treated at two Florida hospitals

Prospectively maintained clinical databases were retrospectively reviewed to identify males and females aged 18 and above who self‐reported as Black or NHW and presented to the Gastrointestinal Clinic at the University of Florida Health Sciences Center (UF; Gainesville) or Moffitt Cancer Center (Moffitt; Tampa) with a strong clinical suspicion or diagnosis of PC between the years 2004 and 2014. These individuals provided written informed consent for biospecimens and data to be donated for research through Institutional Review Board‐approved protocols. Diagnostic confirmation was made using histology from tissue biopsies or surgical resection. Demographic, clinical, epidemiologic, and follow‐up data were collected from the medical record and cancer registry. Additionally, for purposes of comparison, we assessed measures of cachexia among a cohort of Black and NHW “healthy controls” who underwent non‐oncologic surgery at UF.

Myopenia was assessed in a blinded fashion for each cohort using axial CT image analysis.^38^ The combined area of both psoas muscles was measured at the caudal level of the L3 vertebra with both transverse processes visible using previously described methods.^40^, ^46^ Psoas muscle area was then divided by height squared to estimate PMI (cm^2^/m^2^). At UF, the percent difference between the PMI of healthy controls and the PMI of PC cases was also determined as follows: (preoperative PMI in cases—preoperative PMI in controls)/preoperative PMI in controls.

Total skeletal muscle was also measured at the caudal L3 level and included areas of all visible skeletal muscles such as the rectus abdominis; internal, external, and lateral obliques; psoas; quadratus lumborum; and erector spinae muscles. The threshold for this area included hounsfield unit values of −29‐150 which correspond to skeletal muscle. Total skeletal muscle area at L3 was divided by height squared to yield another measure of myopenia, skeletal muscle index (SMI) (cm^2^/m^2^). AW Server version 2.0 (GE Healthcare, Waukesha WI) was used for postprocessing. To classify individuals as myopenic or not myopenic, we used validated gender specific cutoffs of PMI 47(<4.15 cm^2^/m^2^ for women and <5.64 cm^2^/m^2^ for men) and gender‐ and BMI‐specific cut‐points of SMI^48^ (<43 cm^2^/m^2^ for men (BMI < 25 kg/m^2^, <53 cm^2^/m^2^ for overweight/obese men and <41 cm^2^/m^2^ for women). Percent change in musculature over time was calculated for cases as follows: (baseline minus follow‐up muscle index)/baseline muscle index. Descriptive statistics were calculated using frequencies and percents for categorical variables and means and standard deviations for continuous variables.

## 3 RESULTS

### 3.1 Blacks have the highest PC incidence and mortality rates in Florida

FCDS data^44^ revealed that Blacks had higher mean age‐adjusted PC incidence (12.5/100 000) and mortality rates (10.97/100 000) compared to NHW (incidence: 11.23/100 000; mortality:10.27/100 000) and H/L (incidence: 9.62/100 000; mortality: 8.67/100 000), mimicking national estimates (Table 1).^10^ Overall, male Floridians of all races have a significantly higher (*P* < 0.001) PC burden than female Floridians of all races, with mean incidence rates of 13.20 versus 9.85 per 100 000 and mortality rates of 11.68 versus 8.47 per 100 000, respectively. Among males, mean PC incidence and mortality rates were not significantly different among Blacks compared to NHW. Black female Floridians, however, had significantly higher incidence (11.69 vs 9.57, *P* < 0.001) and mortality rates (10.02 vs 8.67, *P* = 0.002) than NHW women. H/L men and women in Florida have significantly lower incidence and mortality rates than NHW (*P* < 0.001). Race/ethnicity and gender‐specific trends in PC incidence and mortality over time suggest these rates are not improving (Figure S1).

**Table 1:** Age‐adjusted pancreatic cancer incidence and mortality rates (per 100 000 persons) in Florida from 2004 to 2014, by race/ethnicity and gender

	Mean	Std Dev	*P*‐Value	2004	2005	2006	2007	2008	2009	2010	2011	2012	2013	2014
All races incidence	11.40	0.43	—	10.69	11.36	11.16	10.91	11.27	11.50	11.27	11.64	12.10	12.09	11.38
NHW incidence	11.23	0.54	Ref	10.49	10.97	11.09	10.62	10.98	11.23	11.13	11.63	12.02	12.24	11.20
Black incidence	12.50	0.78	0.002	12.37	13.04	11.03	12.09	12.70	12.37	13.36	12.69	13.39	11.30	13.13
H/L incidence	9.62	0.77	<0.001	9.75	9.58	10.10	8.71	8.57	10.15	9.57	10.67	9.61	10.62	8.47
All races mortality	9.94	0.39	—	9.28	9.65	9.62	9.54	9.97	9.88	10.24	10.15	10.59	10.08	10.37
NHW mortality	10.27	0.31	Ref	9.64	9.99	10.17	10.03	10.64	10.63	10.55	10.31	10.53	10.14	10.32
Black mortality	10.97	0.62	0.011	12.17	10.70	10.50	10.58	10.92	10.40	12.05	11.20	11.06	10.66	10.46
H/L mortality	8.67	0.44	<0.001	8.51	9.05	9.08	8.30	7.79	8.27	8.63	8.89	9.36	8.65	8.80
All races male incidence	13.20	0.50	Ref	12.70	13.06	12.93	12.84	12.93	13.66	12.73	13.12	14.10	14.05	13.08
All races female incidence	9.85	0.44	<0.001	9.00	9.89	9.63	9.30	9.88	9.62	9.97	10.35	10.35	10.41	9.93
NHW male incidence	13.14	0.61	Ref	12.55	12.66	12.92	12.51	12.75	13.53	12.66	13.26	14.03	14.32	13.33
Black male incidence	13.49	1.25	0.476	14.15	15.26	12.10	14.27	13.58	13.59	13.96	12.85	15.17	11.69	11.80
H/L male incidence	11.60	0.92	0.001	12.95	10.67	12.26	11.29	10.75	12.60	11.35	11.98	10.91	12.61	10.24
NHW female incidence	9.57	0.54	Ref	8.71	9.49	9.49	8.99	9.46	9.19	9.74	10.18	10.25	10.42	9.36
Black female incidence	11.69	1.10	<0.001	11.16	11.60	10.05	10.50	11.76	11.43	12.53	12.65	11.81	11.00	14.04
H/L female incidence	8.51	0.79	<0.001	7.64	9.30	8.57	7.47	7.86	9.42	8.68	9.35	8.79	9.17	7.40
All races male mortality	11.68	0.47	Ref	10.96	11.40	11.30	11.46	11.78	11.72	11.89	11.41	12.62	11.62	12.28
All races female mortality	8.47	0.40	<0.001	7.87	8.16	8.22	7.94	8.50	8.33	8.84	9.05	8.83	8.75	8.74
NHW male mortality	12.08	0.41	Ref	11.52	11.87	11.91	11.99	12.28	12.92	12.08	11.76	12.67	11.72	12.16
Black male mortality	12.20	1.06	0.753	11.78	13.55	11.73	12.08	12.34	10.40	14.14	11.96	13.18	11.74	11.28
H/L male mortality	10.16	0.68	<0.001	10.90	10.05	11.41	9.40	9.54	9.46	10.59	9.91	10.26	9.52	10.75
NHW female mortality	8.67	0.36	Ref	8.05	8.34	8.66	8.29	9.18	8.65	9.20	9.01	8.62	8.73	8.66
Black female mortality	10.02	0.92	0.002	12.21	8.78	9.48	9.38	9.83	10.54	10.26	10.82	9.51	9.77	9.62
H/L female mortality	7.22	0.67	<0.001	6.36	8.29	7.11	7.34	6.20	6.97	6.78	7.78	8.12	7.47	6.97

Abbreviations: H/L, Hispanic/Latino; NHW, Non‐Hispanic White.

### 3.2 Epidemiologic and socioeconomic factors vary by race and ethnicity among PC cases in Florida

FCDS data^44^ showed that the mean age at PC diagnosis was significantly younger among Blacks (67 years) and H/L (69 years) compared to NHW (72 years) (*P* < 0.001). AHCA data^45^ also showed that Black and H/L PC patients tended to be younger at the age of PC discharge (a proxy for age at diagnosis) than NHW counterparts for both genders (Table S1). At diagnosis, a slightly higher percentage of Black PC cases (13.2%) were current smokers compared to NHW (12.3%) and H/L (10.0%) PC cases.^43^ Black Floridians with PC were more likely to be single/unmarried compared to NHW and H/L PC cases (25% vs 12% vs 17%, respectively). Blacks (16%) and H/L (14%) were more likely to be insured with Medicaid than NHW (7%) (Table S2).

### 3.3 Geographic and treatment disparities as contributors to racial/ethnic differences in PC burden

**Figure 1:**
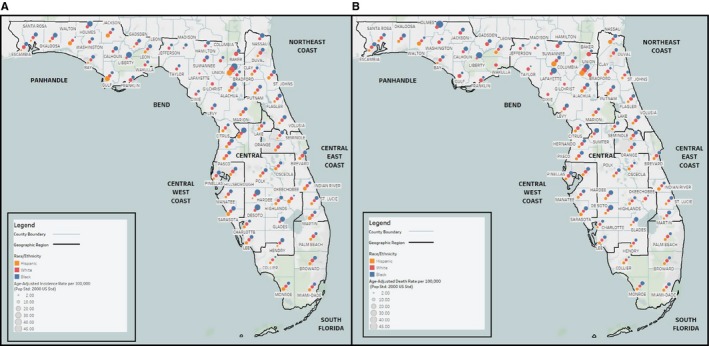
Map of Pancreatic Cancer Incidence and Death Rates by Race/Ethnicity and Florida County. This map displays mean age‐adjusted A. incidence and B. death rates of pancreatic cancer for the years 2004‐2014 (pop std: 2000 US Standard) by Florida county for White, Black, and H/L Floridians (as reported by the Florida Department of Health, Bureau of Vital Statistics). Race/ethnicity groups are distinguished using red (White), blue (Black), or gold (H/L) dots of graduated sizes. Larger dots indicate higher average incidence or death rates. The peninsula is divided into seven geographic regions that were determined by Rosemurgy et al^18^ using the cost of living index and the urban to rural population ratio. H/L, Hispanic/Latino

We evaluated county‐level incidence and mortality data for the seven geographic regions of the peninsula (Panhandle, Bend, Northeast Coast, Central East Coast, Central West Coast, Central, and South) whose borders were previously defined based on the cost of living index (a normalized average that accounts for cost of living across regions) and the urban‐to‐rural population ratio.^18^ Blacks had significantly higher (*P* < 0.05) PC incidence rates than NHW and H/L in the Central East Coast (St. Lucie county), Central West Coast (Sarasota and Pinellas counties), Central (Sumter and Desoto counties), and Panhandle regions (Leon county) (Figure 1A and Table S3). The highest mortality rates among Blacks were observed in the Panhandle (Holmes and Liberty counties), Central Florida (Desoto, Glades, Hardee, and Sumter counties), and the Bend (Lafayette and Baker counties) (Figure 1B and Table S3). Regions having multiple counties with significantly lower PC incidence rates among H/L (compared to NHW) included South Florida, the Northeast, and the Central East and Central West Coasts.

Hospital‐level inpatient data^45^ revealed 189 Florida hospitals that treated patients with a primary discharge diagnosis of PC from 2012 to 2014. Figure S2 illustrates the total number of PC discharges in 2014 (n = 3456) stratified by hospital volume (higher volume defined as ≥25 PC discharges/year and lower volume as <25 PC discharges/year) and race/ethnicity and shows: in both high‐ and low‐volume hospitals, >60% of PC discharges in Florida are among NHW; Blacks account for approximately 11% of PC discharges in the state, 12% of discharges at higher volume hospitals, and 10% of discharges at lower volume hospitals; and H/L account for 16%‐18% of PC discharges overall and at high‐volume hospitals, and 12% of PC discharges at lower volume hospitals.

At the patient level, 14.9% of Blacks underwent pancreatic surgery compared to 17.8% of NHW and 19.0% of H/L.^43^ The most common reasons for Blacks not receiving surgical treatment included: surgery was not part of the first‐course treatment (71.4%), likely because 75% of Black PC cases had regional or metastatic disease; and/or comorbidities (4.6%). For less than 2% of Black PC cases and similar percentages of NHW and H/L cases, surgery was recommended by the physician but was not performed due to patient or family refusal. Hospitals that provided surgical treatment for the highest proportion of Black PC cases are in the South (45%), Northeast (15%), Central (15%), and Central West (14%) regions.^45^ Hospitals that surgically treated the highest number of H/L PC cases are in the South (56%), Central West (17%), and Central (11%) regions.

### 3.4 Radiologic and molecular markers of cachexia may underlie PC racial disparities

**Figure 2:**
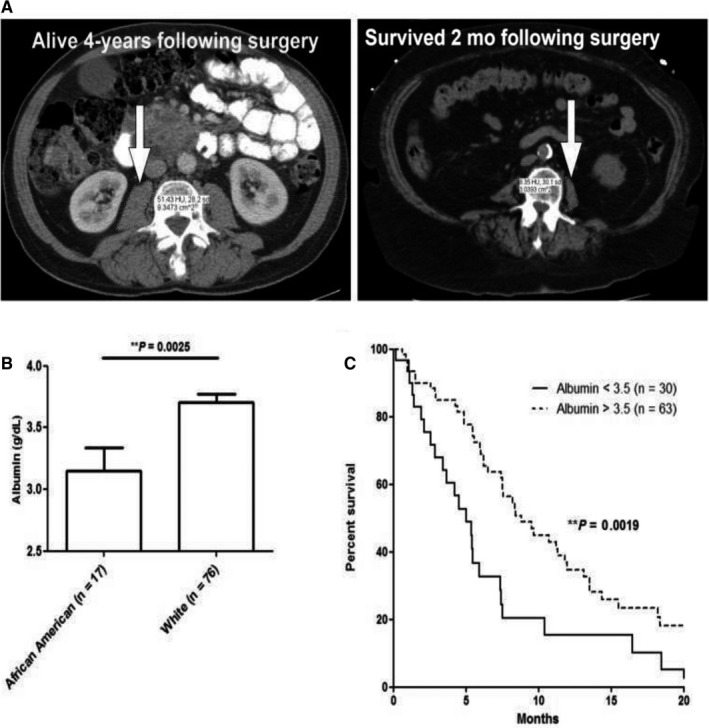
A, Psoas musculature (arrows) is observed for a representative NHW PC patient with long‐term survival (left) and a Black patient succumbing to disease within 2 mo of resection (right). B and C, In a cohort of 93 advanced PC patients, Black patients presented with significantly reduced serum albumin levels (left panel B), which corresponded to poorer survival in Black (solid line) versus NHW patients (dashed line) (right panel C). NHW, Non‐Hispanic White; PC, pancreatic cancer

Next, we evaluated biological correlates of cancer cachexia for Blacks and NHW treated at two Florida cancer centers. Preoperative CT images and serum albumin values were available for a retrospective cohort of 17 Black and 76 NHW PC cases matched on gender, age‐group, and clinical stage surgically treated at the UF Health Sciences Center (Gainesville, Bend Region). Figure 2A displays preoperative CT images for a representative NHW PC patient with high psoas muscle area and longer term survival and a Black patient with low psoas muscle area succumbing to disease within 2 months of resection. Blacks presented with lower mean serum albumin levels compared to NHW, and those with albumin levels <3.5 had reduced survival compared to those with levels >3.5 g/dL (Figure 2B,C).

For a cohort of 20 Black and 34 NHW “control” patients with benign non‐oncologic disease matched to PC cases on gender, age group, BMI, and Charlson Co‐morbidity Score, Black control patients were found to have a significantly greater preoperative PMI than NHW patients (6.65 cm^2^/m^2^ vs 5.20 cm^2^/m^2^, *P* = 0.008). There was no correlation between BMI and PMI (*r* = 0.01900, *P* = 0.320). Serum albumin levels in control patients were similar (3.9 gm/dL vs 4.0 gm/dL, *P* = 0.551), and there was no correlation between albumin and PMI (*r* = 0.0133, *P* = 0.457). Black patients with PC had a significantly lower PMI than Black controls (4.99 cm^2^/m^2^ vs 6.65 cm^2^/m^2^, *P* = 0.007) while the PMI of NHW patients with PC was similar to the index in NHW healthy controls (4.68 cm^2^/m^2^ vs 5.20 cm^2^/m^2^, *P* = 0.294). The calculated per cent differences in PMI between PC cases and controls revealed lower PMI between both Black and NHW at 25.0% and 10.1%, respectively. Figure S3 highlights the significantly greater decrease in PMI among Black PC cases.

**Figure 3:**
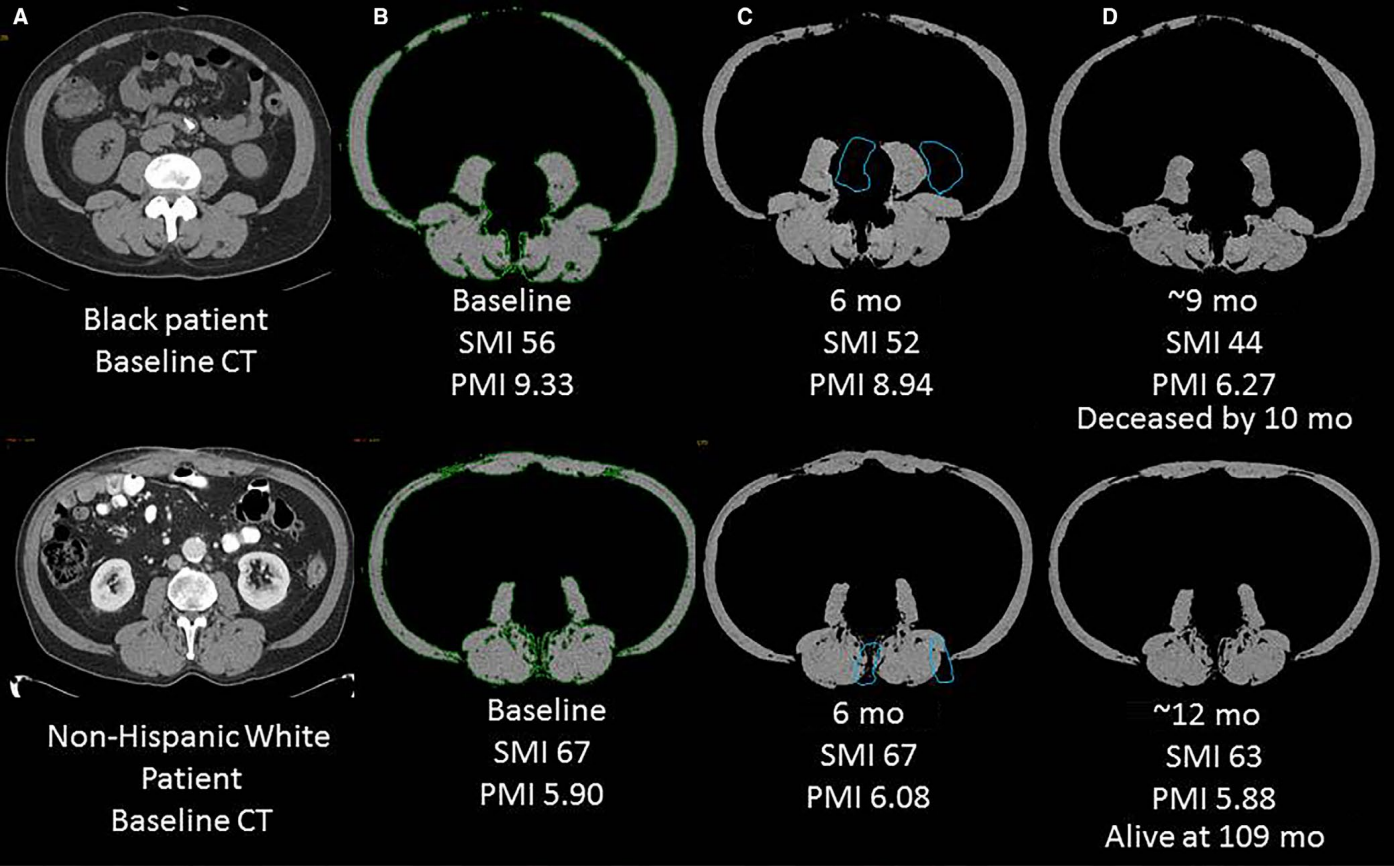
Axial CT images for a Black (top panel) and NHW PC patient (bottom panel). A, Baseline/preoperative image at the inferior endplate of L3 vertebral body. B, Axial image after skeletal muscle is segmented (in green) and non‐muscular structures are excluded using a HU threshold of −29‐+150. C and D, six and 12 mo postoperative follow‐up CT exams showed downward trends in SMI and PMI, especially for the Black patient. Psoas muscle area is outlined in blue on images. Skeletal muscle and psoas muscle areas were calculated on each exam and indexed for patient's height. Note: The NHW patient has higher baseline SMI but lower PMI than the Black patient which can be explained by the relative smaller size of psoas muscles with respect to other skeletal muscles. CT, computed tomography; HU, hounsfield unit; NHW, Non‐Hispanic White; PC, pancreatic cancer; PMI, psoas muscle index; SMI, skeletal muscle index

To assess changes in muscle mass over time, we identified a retrospective cohort of 19 Black PC cases and 19 NHW cases matched on gender, age‐group, and clinical stage diagnosed and treated at Moffitt Cancer Center (Tampa, Central West Coast Region) who had a longitudinal series of CT scans (baseline/pretreatment and follow‐up) available. Compared to NHW cases, Black PC cases of both genders were diagnosed younger and had poorer mean survival (Table 2), in line with national^10^ and state data.^43^ Also consistent with published data,^41^ at baseline, Blacks had slightly higher total skeletal muscle volumes than NHW. Among males, but not females, Blacks had higher psoas muscle volumes than NHW. Psoas musculature decreased over the course of time, with a mean per cent loss of 10.5% among Blacks and 4.8% among NHW. Stratified analyses by gender revealed that per cent loss of PMI and SMI was highest in men (Table 2). The per cent decrease in psoas muscle over time was 24.3% in Black males versus 13.7% in NHW males, and 6.8% in Black females compared to 2.2% in NHW females (Figure S4). Serum albumin levels were lower in Blacks compared to NHW (3.8 gm/dL vs 4.0 gm/dL), and platelet counts were higher in Blacks versus NHW (332.8 vs 268.7 k/UL, respectively) (Table 2). CT images are featured in Figure 3 for two males diagnosed with PC in their early 60's, one Black and one NHW, both of whom were overweight or obese, hypertensive, had prior smoking histories, and underwent surgical resection. Figure 3 shows a greater percentage of psoas muscle loss for the Black man who died by 10 months compared to the NHW man who is still alive (32.8% vs 2.2% loss). In addition to having lower baseline albumin levels compared to the NHW man (3.7 vs 4.4 gm/dL), the Black man had higher platelet counts (324 vs 151 k/UL).

**Table 2:** Selected characteristics of the Moffitt PC cohort by race/ethnicity and gender

	Blacks			Non‐Hispanic White (NHW)		
	Male (n = 4)	Female (n = 15)	Total (N = 19)	Male (n = 4)	Female (n = 14)^a^	Total (N = 18)
Age at diagnosis, mean (range)	55 (33‐75)	60.8 (38‐79)	59.6 (33‐79)	60.5 (51‐75)	65.6 (53‐80)	64.5 (51‐80)
Survival time in months, mean (range)	10.3 (4‐18)	35.1 (8‐88)	29.9 (4‐88)	96 (11‐139)	37.1 (8‐109)	50.7 (8‐139)
Total skeletal muscle volume at baseline (cm^3^), mean (SD)	53.8 (7.7)	31.3 (8.8)	36.1 (12.6)	52.3 (7.1)	30.1 (4.8)	35.0 (10.8)
Total psoas muscle volume at baseline (cm^3^), mean (SD)	8.5 (2.4)	3.38 (1.5)	4.5 (2.8)	7.5 (2.7)	3.7 (1.3)	4.5 (2.6)
Body mass index (BMI) at diagnosis, mean (SD)	24.5 (6.7)	26.9 (7.2)	26.6 (7.0)	24.9 (2.3)	28.2 (7.8)	27.5 (7.1)
Skeletal muscle index (SMI) at diagnosis (cm^2^/m^2^), mean (SD)	54.0 (8.3)	41.2 (5.2)	43.8 (7.8))	54.0 (9.8)	39.5 (6.6)	42.7 (9.5)
Psoas muscle index (PMI) at diagnosis (cm^2^/m^2^), mean (SD)	8.6 (3.5)	4.42 (1.4)	5.3 (2.6)	7.7 (3.0)	4.9 (1.6)	5.5 (2.2)
Prevalence of myopenia at diagnosis (using PMI), N (%)^b^	2 (50)	13 (87)	15 (79)	3 (75)	11 (79)	14 (78)
Time from diagnosis to first follow‐up scan in months, mean SD	5.2 (2.1)	5.6 (2.6)	5.6 (2.5)	5.2 (2.1)	5.6 (3.8)	5.7 (3.3)
Prevalence of myopenia at first follow‐up scan (using PMI), N (%)^b^	2 (50)	6 (32)	15 (79)	3 (75)	10 (56)	14 (78)
Per cent decrease in psoas muscle over time (mean, SD)	24.3 (14.1)	6.8 (2.3)	10.5 (23)	13.7 (20.5)	2.2 (28)	4.8 (27)
Pre‐treatment serum albumin levels (gm/dL), mean (SD)	3.4 (0.5)	3.9 (0.4)	3.8 (0.4)	4.4 (0.2)	3.8 (0.8)	4.0 (0.7)
Pre‐treatment platelet count × 10^3^ UL, mean (SD)	396 (102)	323 (123)	332.8 (120.7)	186 (78.4)	298 (209)	268.7 (187.9)
Clinical tumor size (cm), mean (SD)	4.9 (4.8)	3.4 (1.7)	3.8 (2.6)	2.3 (0.35)	3.6 (1.5)	3.4 (1.5)

a One NHW female case was excluded due to insufficient quality of one or more CT scans.

b Myopenia was defined as PMI < 4.15 cm^2^/m^2^ for women and <5.64 cm^2^/m^2^ for men based on validated thresholds.

## 4 DISCUSSION

A large, high‐quality cancer registry was used to assess PC burden in Florida, a state with racial/ethnic, geographical, and economic diversity.^17^ The highest incidence and mortality rates were observed among Blacks followed by NHW and H/L, in line with data from the Surveillance, Epidemiology, and End Results Program.^49^ Differences were observed by age and insurance type, with Blacks and H/L being younger at diagnosis/discharge and more likely to have Medicaid than NHW, consistent with data outside Florida.^14^, ^50^ Regional differences in PC mortality rates were observed, with higher rates among Blacks compared to NHW in the Central, Bend, and Panhandle regions. Notably, the Panhandle was previously shown to have the highest in‐hospital PC mortality rates in Florida.^18^ At lower volume hospitals, Blacks accounted for 10% of PC discharges whereas NHW and H/L accounted for 72% and 12% of discharges. Thus, Blacks represent less of Florida's population than other groups but have the highest incidence and mortality from PC and the lowest hospital discharge, a proxy measure for treatment or access to care.

Surgery offers the best chance for survival, especially if performed at a high‐volume hospital but only 15%‐20% of PC cases are candidates for surgery.^16^ Black Floridians with PC were the least likely to undergo surgery due to regional or metastatic disease at presentation. In contrast, H/L were most likely to undergo surgery, possibly explaining the lower mortality rates. This finding of surgical treatment being less common among Black patients and most common among H/L is consistent with a recent publication based on a retrospective analysis of PC cases from the National Cancer Database (NCDB)^51^ and with older studies.^6^ However, in contrast to the recent study of NCDB data in which 5% of Black cases and 4% of NHW cases refused surgery that was offered to them,^51^ patient refusal of surgery occurred in less than 2% of Black Floridians and a similar percentage of NHW Floridians. Florida hospitals that surgically treated the highest proportion of Black PC patients were located in the South, Northeast, Central, and Central West regions, while most H/L PC cases were treated in the South, Central West, and Central regions. All of the aforementioned regions except for Central Florida have been associated with having a significant proportion of pancreaticoduodenectomies performed by high‐volume pancreatic surgeons, resulting in lower in hospital mortality, length of stay, and total hospital costs compared to other regions, especially the Panhandle.^18^ Taken together, although a smaller percentage of Black PC patients undergo surgery compared to NHW and H/L, disparities affecting Black Floridians do not seem to be driven by treatment at lower volume hospitals or by lower volume pancreatic surgeons except in rural geographical regions. Large‐scale studies that integrate other epidemiologic and socioeconomic factors, comorbidities, and treatment details to conduct survival analyses are needed to expand upon these findings. Such analyses will be conducted once a more comprehensive dataset that has been requested by the authors is available from the FCDS and the Florida Department of Health.

To study and target modifiable biological factors that may contribute to disparities, we generated the first preliminary data we are aware of to suggest Blacks may be at higher risk for developing advanced stages of cancer cachexia and early demise. Using objective measures of inferior nutritional and functional status, we show that decreases in psoas musculature over time were more pronounced among Blacks compared to NHW and that lower levels of albumin and higher levels of platelets were more commonly observed among Black males. Notably, we also show that Black patients suffer substantially more muscle wasting than NHW patients at diagnosis, even after matching for clinical stage. Platelets are markers of inflammatory and immunologic responses associated with pancreatic carcinogenesis, cachexia, and poor prognosis.^35^, ^36^ It has been postulated that platelets may improve survival of tumor cells by covering and protecting them from stress or immune cells in the bloodstream or by stimulating epithelial‐to‐mesencymal transition.^52^ Thus, therapeutic combinations that include nutritional support and platelet inhibitors and anticoagulants may therefore prove beneficial for certain patient subgroups and warrant further investigation.^53^

Although promising, the cachexia‐related analyses have several limitations. First, these analyses were conducted using data from a retrospective series of PC cases from only two high‐volume cancer centers and may have been influenced by selection bias. Additionally, the small number of cases in each strata precluded multivariate modeling to assess associations with survival while adjusting for comorbidities, smoking history, and socioeconomic variables. Furthermore, this exploratory analysis did not include H/L patients. Despite these limitations, this is first analysis to explore biological correlates of PC cachexia in Blacks and NHW. This analysis would not have been feasible using data from statewide datasets through FCDS and AHCA or regional or national datasets such as SEER, the Cancer Genome Atlas Project, or the Cancer Imaging Atlas Project. Large prospective studies with longitudinal collection of data, images, and biospecimens from racially/ethnically diverse cohorts are needed to confirm and extend upon these speculative findings related to cancer cachexia and other biologically driven hypotheses.

### 4.1 Taking action to minimize disparities

Factors underlying PC disparities reflect the interplay of lifestyle, environmental, socioeconomic, policy, and biological determinants that influence cancer etiology and outcomes (Table S4). Paramount to reducing disparities is addressing socioeconomic and policy barriers and improving health care coordination.^54^ Additionally, it is important to discover mechanisms of carcinogenesis that contribute to observed racial/ethnic disparities.^2^ PC researchers have historically relied on cancer cell lines and mouse models established from and biospecimens donated by NHW PC cases. We have previously shown that patient‐derived cell lines and xenograft models of PC may uncover biological pathways that contribute to the aggressiveness of pancreatic tumors and lead to discovery of effective modalities for earlier diagnosis and prevention of cachexia.^22^, ^55^ As such, our team has started to generate patient‐derived models unique to Blacks and H/L. Additionally, our multi‐institutional transdisciplinary team has established the Florida Pancreas Collaborative, the first state‐wide multi‐center biorepository in the Southeast we are aware of that is dedicated to conducting translational research to reduce disease burden and personalize care for those affected by PC. While initially focused on early detection and prevention efforts,^56^ our collaborative recently expanded to other institutions throughout Florida to develop a stronger foundation to conduct basic, clinical, population‐based, transdisciplinary, and translational disparities research across the PC continuum. The analyses herein underscore rationale for continuing to examine and address PC racial/ethnic disparities in Florida and beyond using integrative approaches that include biologically driven hypotheses and more representative translational research models.

## CONFLICT OF INTEREST

The authors declare no conflicts of interest.

## AUTHORS' CONTRIBUTIONS

Study conceptualization/design: JBP, ACD, DKJ, JWC, JGT; Data curation: JBP, ACD, DKJ, JWC, MEC, JGT; Formal analysis/investigation: JBP, ACD, DKJ, JWC, MEC, JGT; Manuscript writing: JBP, ACD, DKJ, MEC, JGT; Manuscript review/approval: JBP, ACD, DKJ, JWC, MEC, D‐TC, JKT, TEB, JL, BDP, NBK, TJG, KNA, TH, SV, CKG, VNS, PJH, EMC, ARJ, JF, NM, JGT.
