# Supplementary material for: Racial and ethnic disparities in a state‐wide registry of patients with pancreatic cancer and an exploratory investigation of cancer cachexia as a contributor to observed inequities
Source: Cancer Med. 2019 May 9;8(6):3314–24. doi: 10.1002/cam4.2180 (PMC6558500; doi:10.1002/cam4.2180)
Supplement: Supplementary file 1 [file CAM4-8-3314-s001.pdf]

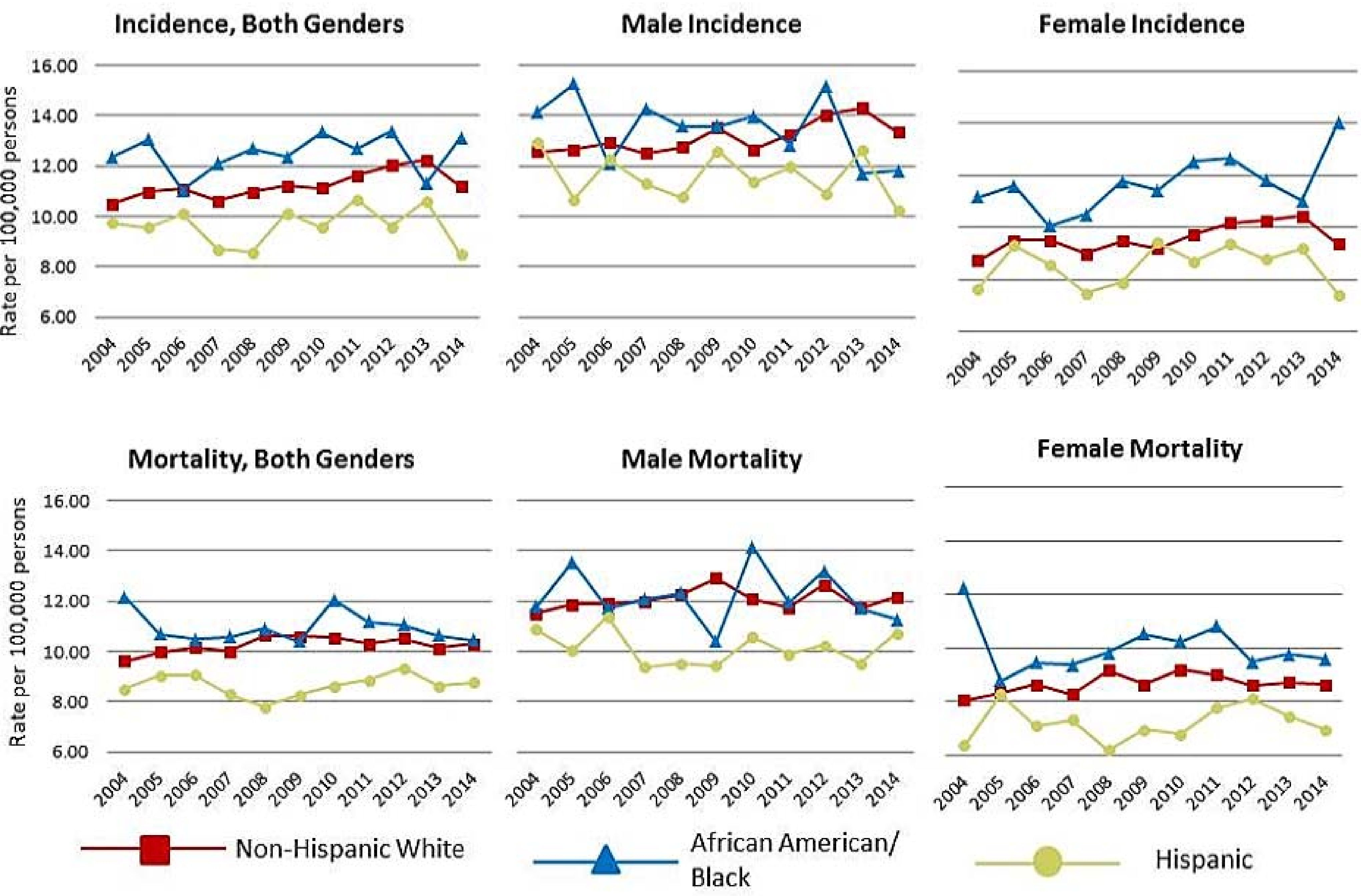

Supplementary Figure 1. Race/ethnicity and gender-specific trends in PC incidence and mortality over time in Florida. These plots display age-adjusted incidence and mortality rates of pancreatic cancer for the years 2004-2014 (pop std: 2000 US Standard) in Florida for White, Black, and H/L (as reported by the Florida Cancer Data System [FCDS]). Race/ethnicity groups are distinguished using red (White), blue (Black), or gold (H/L) lines.
