# Supplementary material for: Racial and ethnic disparities in a state‐wide registry of patients with pancreatic cancer and an exploratory investigation of cancer cachexia as a contributor to observed inequities
Source: Cancer Med. 2019 May 9;8(6):3314–24. doi: 10.1002/cam4.2180 (PMC6558500; doi:10.1002/cam4.2180)
Supplement: Supplementary file 2 [file CAM4-8-3314-s002.pdf]

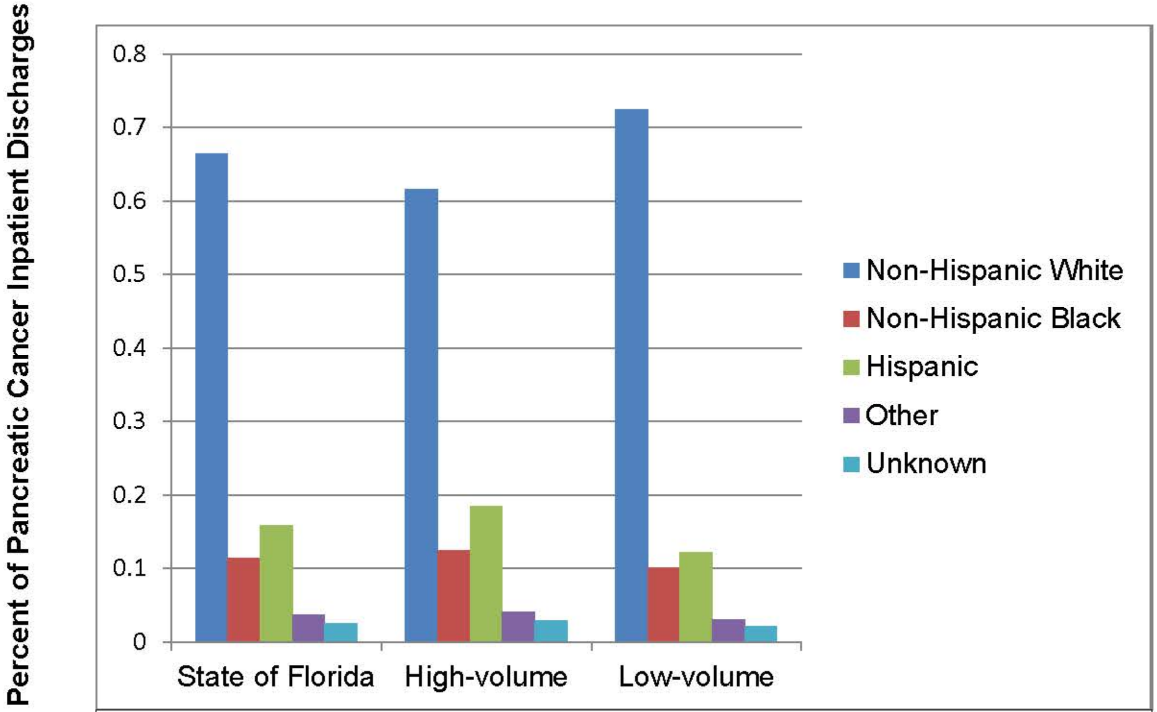

Supplementary Figure 2. Percentage of Pancreatic Cancer Inpatient Discharges by Race/Ethnicity in the State of Florida Overall and at Higher and Lower Volume hospitals. Racial/ethnic distribution of 2014 pancreatic cancer inpatient discharges (based on primary diagnosis codes 140.0-239.9) for the state of Florida (n=3456), higher volume hospitals (≥25 discharges in 2014, n=2025), and lower volume hospitals (<25 discharges in 2014, n=1431) as reported by the Agency for Health Care Administration [AHCA]. Note: The ‘Other’ category includes Asians, Native Americans, and Pacific Islanders.
