# Supplementary material for: Racial and ethnic disparities in a state‐wide registry of patients with pancreatic cancer and an exploratory investigation of cancer cachexia as a contributor to observed inequities
Source: Cancer Med. 2019 May 9;8(6):3314–24. doi: 10.1002/cam4.2180 (PMC6558500; doi:10.1002/cam4.2180)
Supplement: Supplementary file 4 [file CAM4-8-3314-s004.pdf]

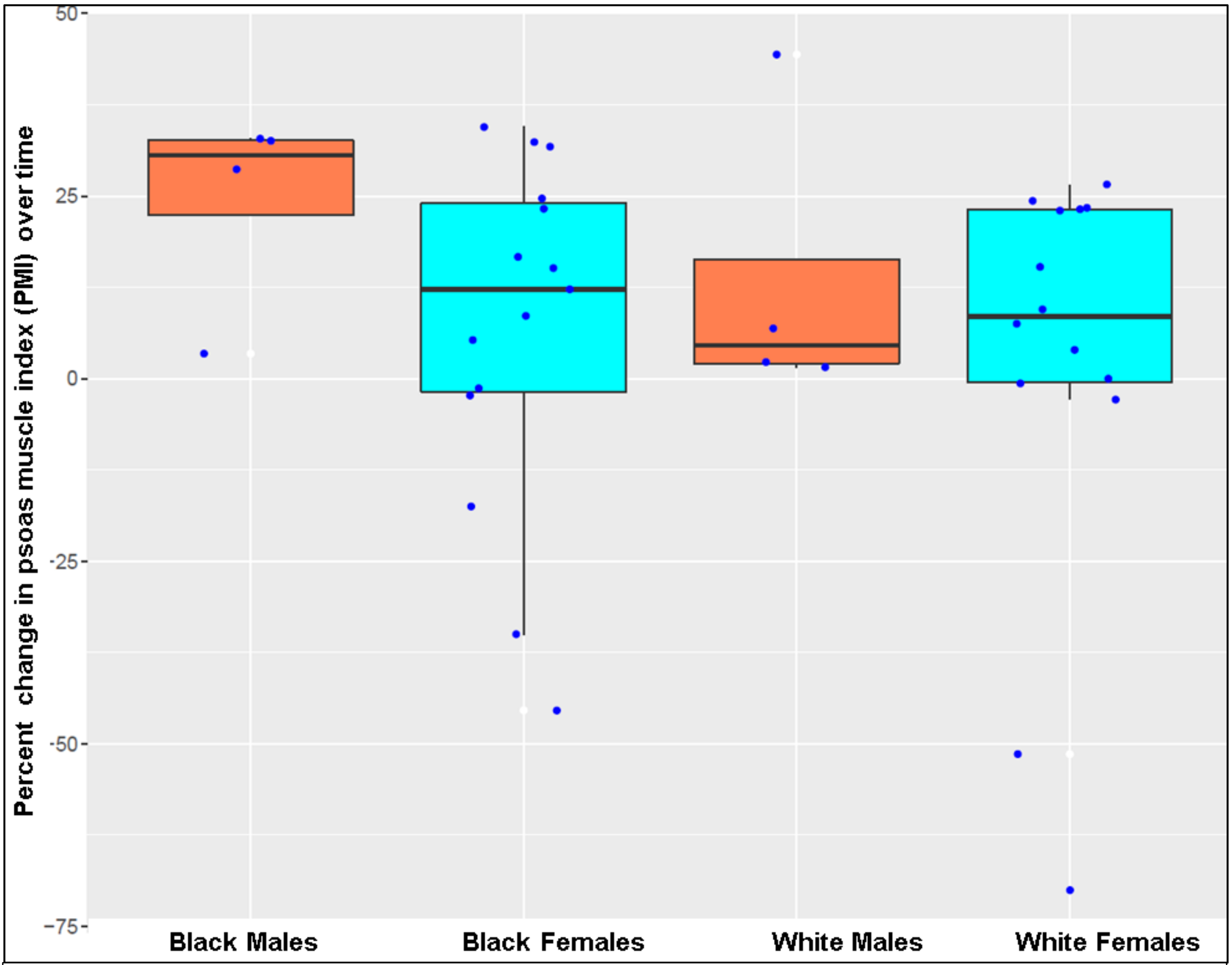


**Supplementary Figure 4. Percent change in psoas muscle index (PMI) from the time of diagnosis to the most recent follow-up scan, by race and gender.** Box plots show general *decreases* in PMI (as depicted by positive percent changes) for PC cases from each strata, though there were some outliers with *gains* in PMI over time (as depicted by negative percent changes).
