# Supplementary material for: Racial and ethnic disparities in a state‐wide registry of patients with pancreatic cancer and an exploratory investigation of cancer cachexia as a contributor to observed inequities
Source: Cancer Med. 2019 May 9;8(6):3314–24. doi: 10.1002/cam4.2180 (PMC6558500; doi:10.1002/cam4.2180)
Supplement: Supplementary file 6 [file CAM4-8-3314-s006.docx]

**Supplementary Table 2. Frequency distribution (count [row percentage]) of the insurance status of pancreatic cancer cases treated in Florida (2014), by race and ethnicity**

|  | **Commercial** | **Government** | **Medicaid** | **Medicaid Managed** | **Medicare** | **Medicare Advantage** | **Other** | **Private Pay** | **Workers Comp** | **Total** |
| --- | --- | --- | --- | --- | --- | --- | --- | --- | --- | --- |
| ***Race*** | | | | | | | | | | |
| **Asian** | 12 (32%) | 3 (8%) | 7 (18%) | 0 (0%) | 1 (3%) | 12 (32%) | 3 (8%) | 0 (0%) | 0 (0%) | 38 |
| **Black** | 94 (23%) | 11 (3%) | 40 (10%) | 24 (6%) | 109 (27%) | 106 (26%) | 12 (3%) | 11 (3%) | 1 (0%) | 408 |
| **Native American** | 2 (40%) | 0 (0%) | 0 (0%) | 0 (0%) | 1 (20%) | 1 (20%) | 1 (20%) | 0 (0%) | 0 (0%) | 5 |
| **Other** | 39 (18%) | 19 (11%) | 23 (11%) | 9 (4%) | 60 (28%) | 52 (24%) | 5 (2%) | 8 (4%) | 0 (0%) | 215 |
| **Pacific Islander** | 0 (0%) | 0 (0%) | 1 (100%) | 0 (0%) | 0 (0%) | 0 (0%) | 0 (0%) | 0 (0%) | 0 (0%) | 1 |
| **Unknown** | 7 (24%) | 1 (3%) | 2 (7%) | 1 (3%) | 9 (31%) | 8 (28%) | 1 (3%) | 0 (0%) | 0 (0%) | 29 |
| **White** | 580 (21%) | 84 (3%) | 125 (5%) | 52 (2%) | 1,174 (43%) | 609 (22%) | 56 (2%) | 57 (2%) | 0 (0%) | 2,737 |
| **Total** | **734 (21%)** | **118 (3%)** | **198 (6%)** | **86 (3%)** | **1,354 (39%)** | **788 (23%)** | **78 (2%)** | **76 (2%)** | **1 (0%)** | **3,433 (100%)** |
| ***Ethnicity*** | | | | | | | | | | |
| **Hispanic/Latino** | 110 (20%) | 25 (5%) | 49 (9%) | 26 (5%) | 140 (25%) | 162 (29%) | 20 (4%) | 23 (4%) | 0 (0%) | 555 |
| **Non-Hispanic** | 606 (22%) | 88 (3%) | 145 (5%) | 60 (2%) | 1,180 (42%) | 604 (22%) | 55 (2%) | 51 (2%) | 1 (0%) | 2,790 |
| **Unknown** | 18 (20%) | 5 (6%) | 4 (5%) | 0 (0%) | 34 (39%) | 22 (25%) | 3 (3%) | 2 (2%) | 0 (0%) | 88 |
| **Total** | **734 (21%)** | **118** | **198 (6%)** | **86 (3%)** | **1,354 (39%)** | **788 (23%)** | **78 (2%)** | **76** | **1** | **3,433 (100%)** |

**Source: 2014 AHCA data**
