# Supplementary material for: Racial and ethnic disparities in a state‐wide registry of patients with pancreatic cancer and an exploratory investigation of cancer cachexia as a contributor to observed inequities
Source: Cancer Med. 2019 May 9;8(6):3314–24. doi: 10.1002/cam4.2180 (PMC6558500; doi:10.1002/cam4.2180)
Supplement: Supplementary file 8 [file CAM4-8-3314-s008.docx]

**Supplementary Table 4. Possible explanations for the disproportionate burden of PC and recommendations for action.**
